# Nitric oxide modulates cardiomyocyte pH control through a biphasic effect on sodium/hydrogen exchanger-1

**DOI:** 10.1093/cvr/cvz311

**Published:** 2019-11-19

**Authors:** Mark A Richards, Jillian N Simon, Ruichong Ma, Aminah A Loonat, Mark J Crabtree, David J Paterson, Richard P Fahlman, Barbara Casadei, Larry Fliegel, Pawel Swietach

**Affiliations:** 1 Department of Physiology, Anatomy and Genetics, Parks Road, Oxford OX1 3PT, UK; 2 Division of Cardiovascular Medicine, Radcliffe Department of Medicine, British Heart Foundation Centre for Research Excellence, John Radcliffe Hospital, Oxford OX3 9DU, UK; 3 Department of Biochemistry, University of Alberta, Edmonton, AB T6G 2H7, Canada

**Keywords:** SLC9A1, NO, Cyclic nucleotides, Heart, pH

## Abstract

**Aims:**

When activated, Na^+^/H^+^ exchanger-1 (NHE1) produces some of the largest ionic fluxes in the heart. NHE1-dependent H^+^ extrusion and Na^+^ entry strongly modulate cardiac physiology through the direct effects of pH on proteins and by influencing intracellular Ca^2+^ handling. To attain an appropriate level of activation, cardiac NHE1 must respond to myocyte-derived cues. Among physiologically important cues is nitric oxide (NO), which regulates a myriad of cardiac functions, but its actions on NHE1 are unclear.

**Methods and results:**

NHE1 activity was measured using pH-sensitive cSNARF1 fluorescence after acid-loading adult ventricular myocytes by an ammonium prepulse solution manoeuvre. NO signalling was manipulated by knockout of its major constitutive synthase nNOS, adenoviral nNOS gene delivery, nNOS inhibition, and application of NO-donors. NHE1 flux was found to be activated by low [NO], but inhibited at high [NO]. These responses involved cGMP-dependent signalling, rather than S-nitros(yl)ation. Stronger cGMP signals, that can inhibit phosphodiesterase enzymes, allowed [cAMP] to rise, as demonstrated by a FRET-based sensor. Inferring from the actions of membrane-permeant analogues, cGMP was determined to activate NHE1, whereas cAMP was inhibitory, which explains the biphasic regulation by NO. Activation of NHE1-dependent Na^+^ influx by low [NO] also increased the frequency of spontaneous Ca^2+^ waves, whereas high [NO] suppressed these aberrant forms of Ca^2+^ signalling.

**Conclusions:**

Physiological levels of NO stimulation increase NHE1 activity, which boosts pH control during acid-disturbances and results in Na^+^-driven cellular Ca^2+^ loading. These responses are positively inotropic but also increase the likelihood of aberrant Ca^2+^ signals, and hence arrhythmia. Stronger NO signals inhibit NHE1, leading to a reversal of the aforementioned effects, ostensibly as a potential cardioprotective intervention to curtail NHE1 overdrive.

## Graphical Abstract

**Graphical Abstract:**
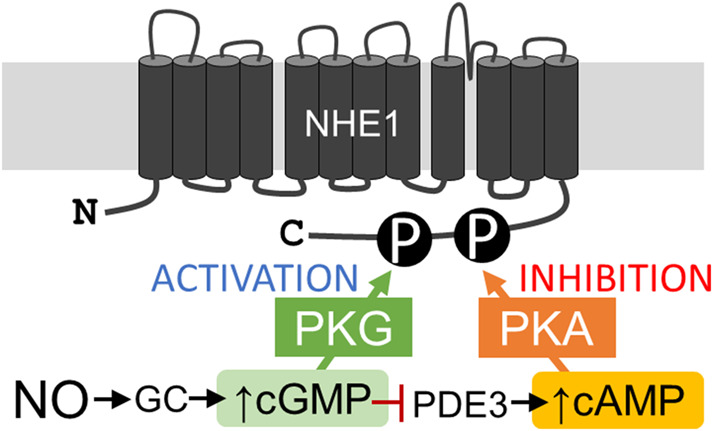

## 1. Introduction

Sarcolemmal Na^+^/H^+^ exchanger-1 (NHE1) is the most powerful regulator of intracellular pH (pH_i_) in the heart, capable of correcting acid–base disturbances within minutes.^1–3^ When fully activated, NHE1 can generate acid-extrusion fluxes of the order of tens of mM/min, coupled to a matching influx of Na^+^ ions.^1–3^ Such high acid-extrusion fluxes are conducive for cardiomyocyte pH_i_ homeostasis, particularly in periods of elevated metabolic acid production, driven by a heightened demand for cardiac work. The importance of maintaining a favourable pH_i_ is perhaps best exemplified by the exquisite pH-sensitivity of contraction, which halves in strength when pH_i_ falls by just 0.2 units.^3–6^

Activated NHE1 can become the largest route of Na^+^ entry into myocytes, meaningfully challenging the corrective capacity of the Na^+^/K^+^ ATPase pump.^7^ An NHE1-evoked rise in intracellular [Na^+^] reduces the driving force for Ca^2+^ extrusion by Na^+^/Ca^2+^ exchange (NCX), leading to an increase in the cellular content of Ca^2+^ ions.^5^^,^^8^^,^^9^ This effect can be positively inotropic,^8^^,^^10^^,^^11^ but beyond a critical level of Ca^2+^ over-load, it may trigger aberrant Ca^2+^ signals, such as Ca^2+^ waves, and hence arrhythmias.^12^^,^^13^ Thus, the cardiomyocyte must carefully balance the need for correcting pH_i_ disturbances against the knock-on effects on Ca^2+^ signalling. This balancing act is exercised by appropriately controlling NHE1 activity.

A physiological trigger for NHE1 activity is intracellular acidity, but the extent to which this evokes a corrective flux depends largely on the post-translational state of the protein.^3^^,^^14^ Various extrinsic factors, including hormones and neurotransmitters, exercise control over NHE1 through kinase-operated cascades.^3^^,^^14^ However, in order for cardiac NHE1 to be responsive to the state of the heart, its regulation must involve auto- or paracrine factors that act locally. Among these factors is nitric oxide (NO), which provides important regulatory cues for a myriad of cardiac pathways.^15–20^ However, there have been conflicting reports of an inhibitory^21^ and activatory effect^22^ of NO on NHE1. Critically, it is unclear how physiologically-relevant levels of NO stimulation affect NHE1. Earlier measurements of [NO] in cardiac tissue indicated levels in the 10–1000 nM range,^23–25^ but more recent analyses have narrowed this to the high picomolar/low-nanomolar range.^26^ Consequently, experimental manoeuvres that had used excessive concentrations of NO donor-substances may not reflect normal physiology.^26^

The major source of NO in the heart is the neuronal-type synthase nNOS,^19^ and its influence on Ca^2+^-handling proteins is well-documented.^15^^,^^20^ NO may affect target-proteins by S-nitros(yl)ation^16^^,^^18^ or through guanylyl cyclase (GC)-dependent cascades.^17^^,^^27^ The latter mechanism canonically triggers cGMP signals, which may secondarily evoke a rise in [cAMP] in cardiomyocytes.^28–30^ Cyclic nucleotides may regulate the fluxes carried by NHE1 by activating kinases that phosphorylate the C-terminus. Previous studies^31–36^ have linked transport activity with two functionally prominent residues, Ser648 and Ser703, predicted to be consensus sites for PKA and PKG^14^. Whereas Ser648 phosphorylation inhibits NHE1 activity,^31^ phosphorylation at Ser703 has the opposite effect.^36^ It is thus unclear how such an apparently ambiguous design would be compatible with finely-controlled oversight by NO.

Here, we studied the effects of NO on cardiac NHE1 activity, interrogated by means of direct functional assays performed on isolated adult ventricular myocytes. Given that NHE1 is electroneutral, its activity cannot be inferred from measurements of membrane currents; instead, the most appropriate readout is the recovery of pH_i_, after accounting for H^+^ buffering.^1^ Such measurements are able to resolve inhibitory or activatory effects on transporter fluxes, and they relate more closely to cardiac physiology. Using manipulations that include nNOS knockout, adenoviral nNOS gene delivery, nNOS inhibition, and pharmacological NO titrations, we demonstrate that low levels of NO activate NHE1, whereas higher doses switch to a net inhibitory effect. These dynamic effects were not dependent upon S-nitros(yl)ation^16^^,^^37^ but, instead, involved a careful balancing of activatory cGMP and inhibitory cAMP signals. We show that PKG and PKA can phosphorylate the activatory Ser703 at the C-terminus of NHE1 *in vitro*, but Ser648, an inhibitory residue, is more strongly phosphorylated by PKA. Finally, we demonstrate how the biphasic modulation of NHE1 by NO affects Ca^2+^ handling, using the example of spontaneous Ca^2+^ waves.

## 2. Methods

### 2.1 Adult ventricular myocyte isolation

Ventricular myocytes were isolated from hearts by enzymatic digestion and mechanical dispersion. This study used myocytes from wild-type male Sprague-Dawley rats (300–325 g), or 4- to 5-month-old nNOS−/− knockout mice and their wild-type littermates, bred under license (PPL 30/3374). nNOS knockout was achieved by targeted disruption of exon 2 of nNOS.^38^ Rats were sacrificed by stunning followed by cervical dislocation, and mice were sacrificed by cervical dislocation and exsanguination in accordance with UK Home Office regulations (Schedule I of A(SP)A 1986), approved by national and University ethics committees. Hearts were rapidly removed and rinsed in isolation solution (in mM): 120 NaCl, 4 KCl, 1.2 MgCl_2_, 11 glucose, 10 HEPES, 2 NaH_2_PO_4_, 2.5 pyruvate pH 7.4 at 37°C; supplemented with heparin sodium (5 units/L), and then mounted on a Langendorff perfusion system. Once cleared of blood, the heart was perfused with collagenase type II (0.9 mg/mL, Lorne Laboratories, UK) and protease type XIV (0.09 mg/mL, Sigma, UK) for 10 min, cut into pieces, triturated with a Pasteur pipette and dissociated cells filtered through a 250-µm nylon mesh.

### 2.2 Neonatal ventricular myocyte isolation and culture

Neonatal rat ventricular myocytes were isolated from 1- to 2-day-old Sprague-Dawley rats. Animals were euthanized by cervical dislocation and exsanguination according to Schedule 1 of the Animals (Scientific Procedures) Acts 1986. Experiments were approved by Oxford University ethical review boards and conform to the guidelines from Directive 2010/63/EU. Cells were isolated from ventricular tissue of excised hearts by enzymatic digestion and plated onto fibronectin-coated Ibidi µ-slides (Ibidi, Germany) then cultured as described previously.^39^

### 2.3 MDA-MB-468 cells

MDA-MB-468 cells, obtained from ATCC, were cultured in DMEM with 10% FCS in an atmosphere of 5% CO_2_. Cells were plated on Ibidi µ-slides (Ibidi, Germany), grown to confluency, and used for superfusion experiments.

### 2.4 Viral transduction of myocytes

Adult ventricular myocytes were infected with adenovirus containing the gene for nNOS under a CMV promoter,^40^^,^^41^ the FRET-based cAMP sensor H187,^42^ or the appropriate sham controls. Myocytes were cultured overnight on µ-slides (Ibidi, Germany) in myocyte culture medium (MEM supplemented with 9 mM NaHCO_3_, 1% L-glutamine, 1% penicillin/streptomycin), 0.5 µM cytochalasin D to preserve cell shape,^43^ and adenovirus (H187 final titer 5 × 10^9^ viral particles/mL, nNOS final titer: 10^7^ viral particles/mL). After overnight culture, the virus-containing medium was replaced with myocyte culture medium supplemented with 2.5% FBS.

### 2.5 NO donors and scavengers

Experiments used either sodium nitroprusside (SNP) or 3-ethyl-3-(ethylaminoethyl)-1-hydroxy-2-oxo-1-triazene (NOC12), which are short and long half-life NO donors, respectively. To clamp [NO] to physiological levels, the NO scavenger CPTIO (2-(4-carboxyphenyl)-4,4,5,5-tetramethylimidazoline-1-oxyl-3-oxide) was combined with NOC12; additionally, 300 µM urate to scavenge NO_2_ and ONOO^−^.^44^

### 2.6 Solutions and superfusion

Solutions were delivered at 37°C to a custom-made Perspex superfusion chamber with a coverslip glass bottom (for experiments on adult myocytes) or an Ibidi µ-slide (for experiments on cultured cells). Normal Tyrode contained (in mM) 135 NaCl, 4.5 KCl, 1 CaCl_2_, 1 MgCl_2_, 11 glucose, 20 Hepes at pH 7.4. In ammonium-containing solutions, NaCl was iso-osmotically replaced with NH_4_Cl. In acetate-containing solutions, NaCl was iso-osmotically replaced with NaAcetate.

### 2.7 Dual microperfusion

The boundary between microstreams was visualized by including 10 mM sucrose into one of the two microstreams. Flows were adjusted to produce a sharp boundary, which was positioned across the middle of a myocyte.^45^^,^^46^

### 2.8 Fluorescence imaging

To image cAMP levels, the FRET-sensor H187 was excited at 405 nm and fluorescence was measured at 480 ± 10 nm and 530 ± 10 nm in *xy*-mode on a Zeiss LSM700 confocal system. To image NO levels, DAR-4M was AM-loaded into cells (10 µM DAR-4M AM, for 10 min), and its fluorescence was excited at 514 nm and emission detected at 580 ± 20 nm on a Leica TCS NT system.^47^ To image Ca^2+^ waves, Fluo3 was AM-loaded into myocytes (5 µM Fluo3, for 10 min), and its fluorescence was excited at 488 nm and detected >520 nm on a Leica TCS NT confocal system in *xt* mode. To measure pH_i_, myocytes were AM-loaded with cSNARF1 (10 µM, for 10 min), and fluorescence was excited at 530 nm and detected simultaneously at 580 ± 10 nm and 640 ± 10 nm on an inverted Olympus microscope with an Orca 05G CCD Camera (Hamamatsu, Japan) and Optosplit (Cairn Research, UK).

### 2.9 Expression of NHE1 C-terminus (His182)

The C-terminus of NHE1 was expressed in a bacterial (BL21-SI) system, as described previously.^48^ Bacteria were cultured in LBON media (bacto tryptone 10 g/L, bacto yeast extract 5 g/L, AMP 100 mg/L) at 30°C. After reaching an optical density of 0.6 (600 nm), expression was induced by adding 300 mM NaCl and incubation for 4 h. His182 was purified as described previously.^48^

See Supplementary material online for *Kinase reactions, Western blotting, Phos-tag gel analysis, S-nitros(yl)ation (iodoTMT) assay, organo-mercury enrichment, antibodies, and mass spectrometry*.

### 2.10 Statistics

Differences tested by two-way ANOVA at 5% significance, followed by *post hoc* test (Fisher’s LSD). Data are reported as means ± SEM, and repeats as ‘number of cells/number of animals’. Flux data are normally distributed continuous variables. For non-normally distributed data comparing two groups, statistical analysis was done using a Mann–Whitney *U*-test.

## 3. Results

### 3.1 PKA and PKG produce distinct patterns of NHE1 phosphorylation

**Figure 1:**
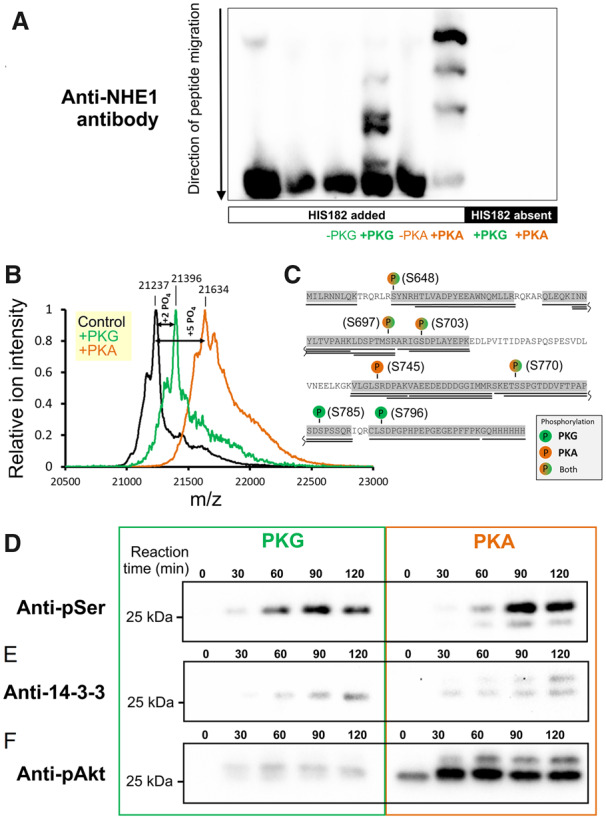
Phosphorylation of the NHE1 C-terminus by PKA and PKG. (*A*) Various combinations of kinase (PKA or PKG; 8 ng/µL) and protein (His182; 20 ng/µL) were reacted *in vitro* and run on a Phos-tag gel to seek evidence for differences in phosphorylation pattern. (*B*) His182 (100 ng/µL) was reacted *in vitro* with PKA (100 ng/µL) or PKG (50 ng/µL) and analysed by LC-MS/MS mass spectrometry for phosphorylation sites. Results indicated a mean phosphorylation stoichiometry of five and two phosphate groups introduced by PKA and PKG, respectively. (*C*) Residues identified as being phosphorylated by PKA and PKG. (*D*) Time course of reaction between His182 (20 ng/µL; plus 500 µM ATP) and either PKG (8 ng/µL; *left*) or PKA (8 ng/µL; *right*)*.* Western blots were performed using an antibody raised against phosphorylated serine residues. (*E*) Western blot performed using antibody against the phosphorylated 14-3-3 binding motif (detecting Ser703 in NHE1). (*F*) Western blot performed using antibody against phosphorylated Akt substrate (Ser648 in NHE1). *Note*: blots for PKA- and PKG-reacted His182 were prepared with the same amount of substrate and developed simultaneously with identical exposure time. See Supplementary material online, *Figure S1* for loading controls.

NO signalling in the myocyte can activate PKG via the canonical pathway involving cGMP^17^^,^^27^ and, under certain conditions, PKA through a rise in [cAMP].^28–30^ To study how NHE1 responds biochemically to these kinases, the C-terminus (wherein most modulatory influences converge) of the human protein was expressed in a bacterial system.^48^ This fragment, thenceforth referred to as His182, was reacted *in vitro* with PKA or PKG, and phosphorylation was analysed by Phos-tag gel blotting (*Figure 1A*). Both PKA and PKG were able to phosphorylate His182, but the mobility shifts were distinct, indicating that the kinases act differentially on NHE1. Two important residues linked directly to regulating NHE1 transport activity,^31^^,^^36^ and predicted substrates for PKA and PKG,^14^ are Ser648 and Ser703. To test if these residues are phosphorylated, kinase reactions were subject to analysis by mass spectrometry (*Figure 1B*). This revealed that His182 was phosphorylated on multiple serine residues, including Ser648 and Ser703. Consistent with the Phos-tag analysis, mass spectrometry revealed a differential pattern of His182 phosphorylation by PKA and PKG (*Figure 1C*). Western blotting using antibodies raised against phosphorylated serine confirmed phosphorylation of His182 by both PKA and PKG, albeit with distinct bands (*Figure 1D*).

To investigate how the phosphorylation status of Ser648 and Ser703 responds to PKA and PKG, antibodies recognizing specific phosphoserine motifs were used for western blotting (see Supplementary material online, *Figure S1* for loading controls). An antibody raised against the 14-3-3 binding motif detected Ser703 phosphorylation by PKA and PKG, albeit with a distinct immunoreactivity pattern (*Figure 1E*). In contrast, an antibody raised against the Akt phosphorylation consensus sequence, i.e. Ser648 on NHE1, showed substantially greater immunoreactivity towards PKA-treated His182, and only weak signal with PKG (*Figure 1F*). These findings indicate that both PKA and PKG can phosphorylate Ser703, which underpins NHE1 activation,^36^ but that PKA is considerably more selective for Ser648, a residue responsible for NHE1 inhibition.^31^ To investigate how these kinases are recruited physiologically by NO to regulate cardiac NHE1, the next experiments measured transport activity in ventricular myocytes subjected to pharmacological or genetic interventions that target PKG- and PKA-mediated cascades.

### 3.2 Basal NO production activates cardiac NHE1

**Figure 2:**
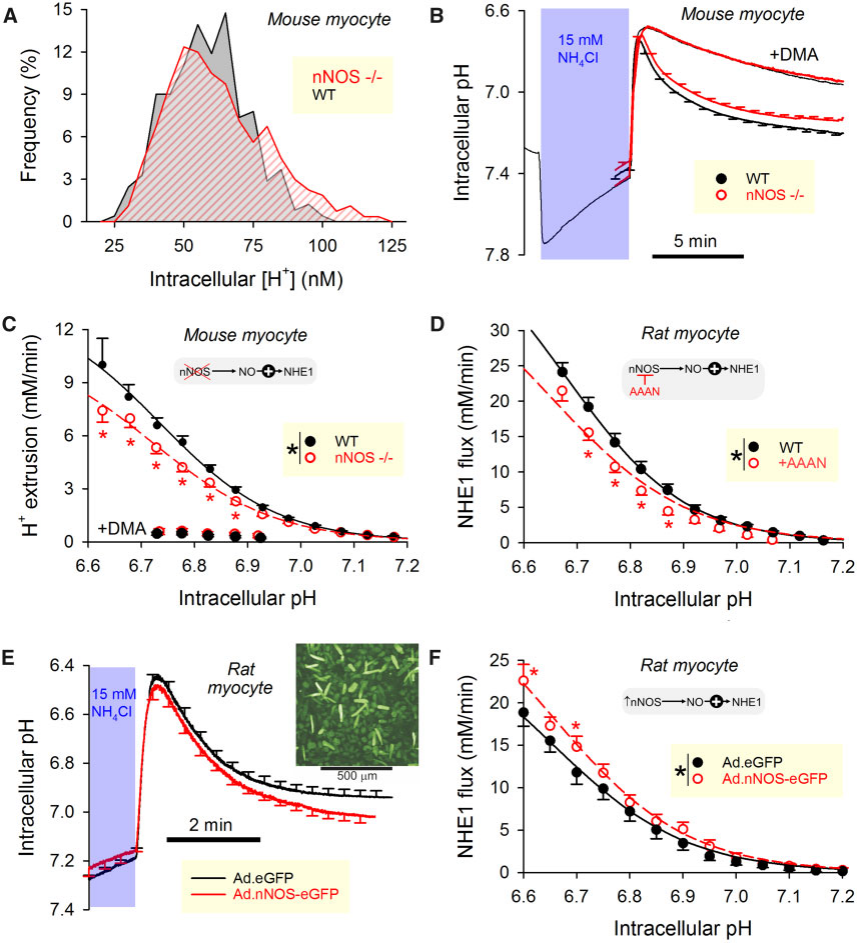
nNOS-derived NO activates NHE1 activity. (*A*) Histogram of resting [H^+^]_i_ in ventricular myocytes isolated from nNOS knockout mice (*N* = 267 from five mice) or their wild-type littermates (*N* = 244 from five mice). (*B*) Time course of ammonium prepulse (mean ± SEM; *N* = 64 from five nNOS knockout mice and *N* = 54 from five wild-type littermates). Hepes-buffered superfusates, 37°C. Solution manoeuvre acid-loaded myocytes to interrogate acid-extrusion fluxes during recovery phase (blocked by 30 µM dimethylamiloride, DMA). (*C*) Flux calculated from the rate of pH_i_ change and buffering capacity (Supplementary material online, *Figure S2*). NHE1-dependent fluxes were slower in nNOS knockout mice (*P* < 0.0001, two-way ANOVA). (*D*) NHE1 flux measured in wild-type rat ventricular myocytes. Hepes-buffered superfusates, 37°C. Treatment with selective nNOS inhibitor AAAN (5 µM; *N* = 16 from five rats) reduced activity relative to control (*N* = 18 from five rats) (*P* < 0.0001, two-way ANOVA). (*E*) Ammonium prepulse performed on adenovirally infected rat ventricular myocytes cultured overnight. Hepes-buffered superfusates, 37°C. Infected cells were identified by eGFP fluorescence (inset). (*F*) NHE1 activity was accelerated (*P* < 0.0001, two-way ANOVA) in myocytes infected with nNOS gene (*N* = 16 cells from four independent infections) compared to sham infected cells (*N* = 28 from five independent infection).

nNOS knockout mice were used to investigate the effect of basal NO production on pH_i_ control by NHE1 (see Supplementary material online, *Figure S2* for confirmation of nNOS gene ablation). Resting pH_i_ was measured in enzymatically isolated cardiomyocytes loaded with the pH reporter dye, cSNARF1, and superfused in physiological CO_2_/${\text{HCO}}_{3}^{-}$-buffered solution. The frequency distribution of resting [H^+^]_i_ in nNOS−/− myocytes was, compared to cells from wild-type littermates, broader and extended into the acidic range. Thus, myocytes lacking basal NO production are less able to defend a favourable (mildly alkaline) pH_i_ (*Figure 2A*). Intrinsic buffering capacity, attributable largely to proteins, was no different between the two experimental groups (Supplementary material online, *Figure S3*; measured by a step-wise NH_4_Cl removal method^49^) To measure NHE1 activity, the transporter was activated by lowering pH_i_, attained by means of an ammonium prepulse solution manoeuvre.^1^ For these experiments, myocytes were superfused with Hepes-buffered solution to inactivate ${\text{HCO}}_{3}^{-}$-dependent transport. Under these conditions, recovery from an acidic pH_i_ is mediated primarily by NHE1, as demonstrated by its sensitivity to dimethylamiloride (DMA; 30 µM), an NHE1 inhibitor (*Figure 2B*). Given that NHE1 is exquisitely sensitive to [H^+^], data were presented as pH_i_-flux curves, and comparisons are made at matching levels of pH_i_. NHE1 flux, calculated as the product of buffering capacity and the rate of pH_i_ change during the recovery phase, was slower in nNOS−/− myocytes compared to wild-type cells (*Figure 2C*), demonstrating that basal NO production has an activatory effect on NHE1. There was no effect of the nNOS knockout on the DMA-insensitive component of flux.

Genetic ablation of nNOS may have exerted its activatory effect through a reversible post-translational effect driven by NO, or through a slower up-regulation of NHE1 protein. These two possible mechanisms were tested by measuring the acute response of NHE1 flux to nNOS inhibition with the compound AAAN.^50^ Wild-type rat myocytes treated with 5 µM AAAN had a left-shifted NHE1-pH_i_ curve (*Figure 2D*), indicating that basal nNOS activity is exerting a reversible, post-translational effect on NHE1.

Further testing of the effect of nNOS activity on NHE1 used rat myocytes infected with an adenovirus encoding for an nNOS-eGFP fusion protein. Cells incubated overnight with virus produced a good yield of construct expression, as determined by eGFP fluorescence (*Figure 2E*, inset). Compared to cells incubated overnight with virus containing only the eGFP gene (i.e. sham infection), adenoviral nNOS gene delivery hastened pH_i_ recovery to a more alkaline resting pH_i_, and increased NHE1 flux (*Figure 2F*). Overall, these findings demonstrate that basal nNOS-derived NO exerts an activatory influence on NHE1.

### 3.3 Exogenously delivered NO exerts a dose-dependent effect on NHE1

**Figure 3:**
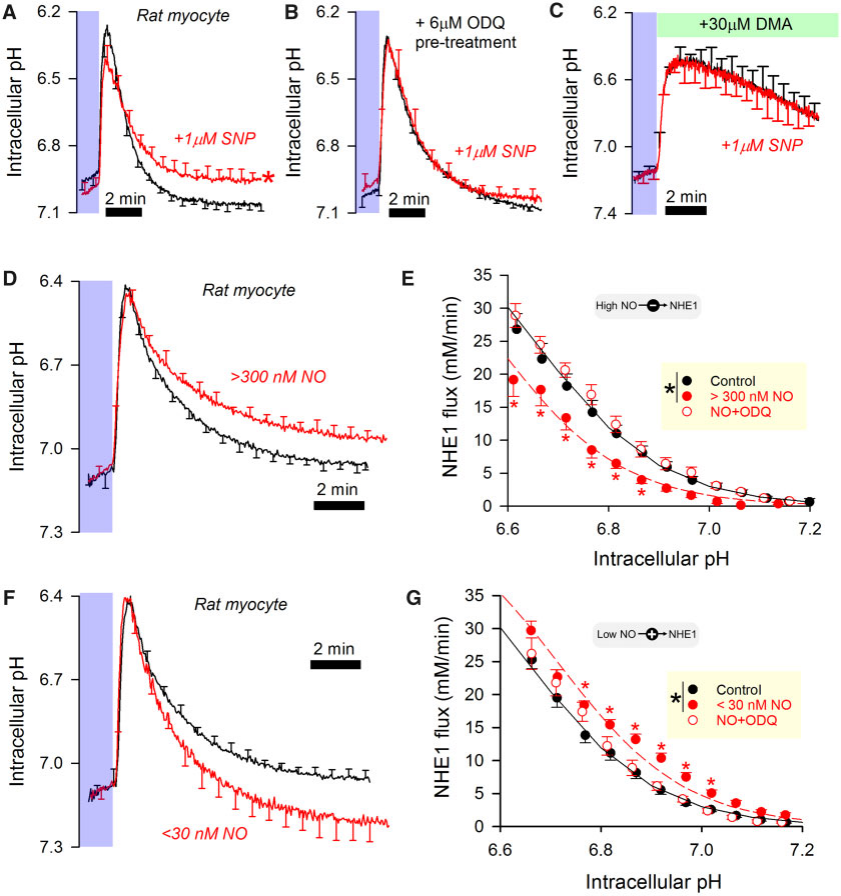
NO delivered from donors produces a concentration-dependent biphasic effect on NHE1 activity. (*A*) Wild-type rat myocytes. Hepes-buffered superfusates, 37°C. The short half-life NO donor, sodium nitroprusside (SNP), was added to solutions 4–6 min before activating NHE1. With 1 µM SNP, NO is expected to peak at 600 nM during pH_i_ recovery (control: *N* = 35 cells from five animals; SNP: *N* = 31 cells from five animals). (*B*) Experiments repeated on myocytes that had been pre-treated with 6 µM ODQ (1H-[1,2,4]Oxadiazolo[4,3-a]quinoxalin-1-one), an inhibitor of guanylyl cyclase (GC; ODQ: *N* = 24 cells from four animals; ODQ+SNP: *N* = 26 cells from four animals). (*C*) CO_2_/${\text{HCO}}_{3}^{-}$-buffered superfusates, 37°C; 30 µM dimethylamiloride (DMA) included to block NHE isoforms. pH_i_ recovery mediated by ${\text{HCO}}_{3}^{-}$ dependent transporters is unaffected by NO (control: *N* = 15 cells from three animals; SNP: *N* = 15 cells from three animals). (*D*) Wild-type rat myocytes. Hepes-buffered superfusates, 37°C. The long half-life NO donor NOC12 was added to solutions 10 min before activating NHE1. At a concentration of 5 µM, NOC12 is expected to maintain NO above 300 nM for the duration of pH_i_ recovery. (*E*) NHE1 fluxes. Control (*N* = 21 from five animals), effect of NOC12 (*N* = 18 from five animals), and effect of NOC12 on ODQ-pretreated cells (*N* = 26 from five animals). Significant difference between control and NOC12 (*P* < 0.0001), but not between control and NOC12+ODQ (*P* = 0.06). (*F*) Lower concentrations of NO (<30 nM) were produced by mixing donor NOC12 (300 µM) with scavenger CPTIO (100 µM). (*G*) NHE1 fluxes. Control (*N* = 21 from five animals), effect of NOC12/CPTIO (*N* = 19 from five animals), and effect of NOC12/CPTIO on ODQ-pretreated cells (*N* = 10 from four animals). Significant difference between control and NOC12 (*P* < 0.0001), but not between control and NOC12/CPTIO+ODQ (*P* = 0.08).

The actions of NO on NHE1 were further studied using an exogenous source of NO delivered by superfusion. SNP has widely been used as a short half-life NO donor, producing substantial NO release peaking near 600 µM within tens of minutes of addition to superfusates (Supplementary material online, *Figure S4*). Recovery from an acid-load (Hepes-buffered superfusates) was first measured under control conditions, and repeated in the presence of SNP (1 µM). Exposing the myocytes to a high concentration of NO resulted in a slowing of pH_i_ recovery and a more acidic resting pH_i_ (*Figure 3A*). This attenuation of NHE1 flux was not due to an NO-independent, time-dependent attrition, because a consecutive pair of control measurements produced a similar recovery (Supplementary material online, *Figure S5*). The inhibitory effect of SNP was absent in cells pre-treated with 1H-[1,2,4]oxadiazolo[4,3-a]quinoxalin-1-one (ODQ; 6 µM), an irreversible inhibitor of GC, indicating the involvement of cGMP signals (*Figure 3B*). These data suggest that a high concentration of NO released from SNP *inhibits* NHE1 flux, in apparent contradiction to the activatory effect of basal NO production by nNOS described earlier (see *Figure 2*). SNP (1 µM) had no effect on Na^+^-HCO_3_^−^ cotransport (NBC), the other major acid-extruder in myocytes, measured in CO_2_/HCO_3_^−^ buffered superfusates containing DMA to block NHE1 (*Figure 3C*).

The actions of NO on NHE1 were investigated further using NOC12, a donor with longer half-life, thus releasing a steadier flow of NO. Kinetic modelling using published stability constants indicates that myocytes superfused with 5 µM NOC12 are exposed to >300 nM NO (Supplementary material online, *Figure S4*). At this concentration, NO exerted an inhibitory effect on the rate of pH_i_ recovery and acidified steady-state pH_i_ (*Figure 3D*), akin to the effect of SNP. The inhibitory effect of 5 µM NOC12, which could be described in terms of a left-shift in the NHE1-pH_i_ curve by ∼0.1 units, was absent in myocytes pretreated with ODQ, confirming that the exogenously released NO was signalling via cGMP (*Figure 3E*). Although many previous studies have used micromolar doses of donors to interrogate NO signalling, the concentration of NO attained may be considered supra-physiological.^26^ To measure the effects of lower NO concentrations, NOC12 was combined with a scavenger, CPTIO, to titrate [NO] down to the low-nM range.^44^ A kinetic model determined that combining 300 µM NOC12 with 100 µM CPTIO (plus 300 µM urate to scavenge the by-product NO_2_) could maintain [NO]<30 nM^27^^,^^44^ (Supplementary material online, *Figure S4*). At this lower [NO], pH_i_ recovery was faster and progressed to a more alkaline resting pH_i_ (*Figure 3F*) indicating NHE1 activation (*Figure 3G*), which is now consistent with the effect of nNOS-derived NO. This effect, observed as a right-shift in the NHE1-pH_i_ curve, was absent in myocytes pretreated with ODQ (*Figure 3G*). Thus, high and low concentrations of NO produce opposite effects on NHE1 activity, yet both involve cGMP signals.

### 3.4 Biphasic modulation of NHE1 by NO is a special property of the adult cardiomyocyte

To determine if the biphasic modulation of NHE1 by NO is a more general property of NHE1-expressing cells, measurements were performed on cultured neonatal rat ventricular myocytes and on a human breast cancer cell line MDA-MB-468, both of which produce NHE1 fluxes that are comparable in magnitude to those in adult ventricular myocytes. However, the biphasic effect of NO on NHE1 was not observed in neonatal myocytes (Supplementary material online, *Figure S6A* and *B*) or MDA-MB-468 cells (Supplementary material online, *Figure S6C* and *D*). These findings argue that NO signals modulate NHE1 in a highly context-sensitive manner, involving elements that are present in adult cardiac myocytes, but not necessarily in other cell types. Consequently, expression systems and *in vitro*-based assays are not appropriate for interrogating the mechanisms of cardiac NO-NHE1 interplay; instead, the relevant cascades must be studied in primary adult myocytes.

### 3.5 NO signals can be transmitted onto remote NHE1 targets and do not involve S-nitros(yl)ation

**Figure 4:**
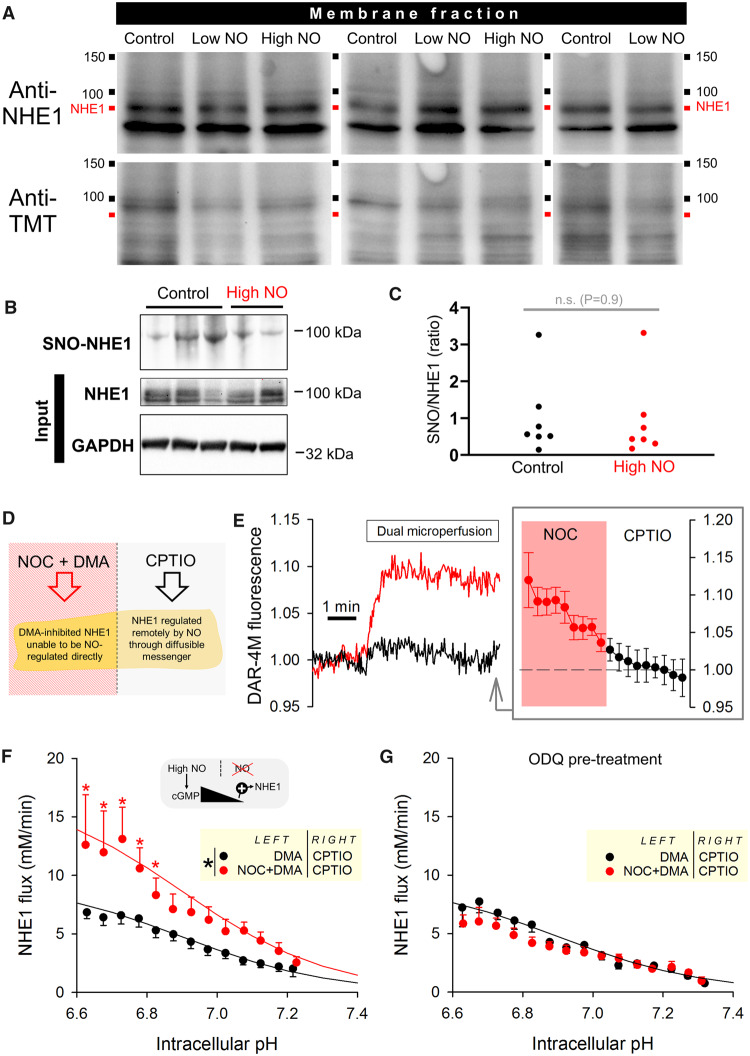
NO regulates NHE1 activity via a diffusible messenger and not via S-nitros(yl)ation. (*A*) Hearts were Langendorff-perfused with normal Tyrode, or Tyrode with high (5 µM NOC12) or low (300 µM NOC12 + 100 µM CPTIO) [NO]. S-nitros(yl)ation assay was performed on membrane fractions of heart lysates. NHE1 staining visible as bands near 90 kDa. Re-probed for TMT as readout of protein S-nitros(yl)ation: no increase in nitros(yl)ation was detectable in the presence of NO donors. (*B*) Direct NHE1 *S*-nitros(yl)ation (SNO) was evaluated by an independent method that uses organo-mercury enrichment. (*C*) No difference in the extent of SNO-enriched NHE1 was observed between control and high NO-treated hearts. *N* = 7 per group; not significant (*P* = 0.9); Mann–Witney *U*-test. (*D*) Dual microperfusion of isolated rat myocyte. Left microstream produced a NO microdomain from slow-releasing NOC12 (5 µM); NHE1 was inactivated locally by dimethylamiloride (30 µM). Right microstream contained CPTIO to scavenge any spill-over of NO from the left microstream. (*E*) Measurement of NO microdomain using fluorescent dye DAR-4M, showing gradient of [NO] along length of cell (*N* = 9 cells from three animals). (*F*) NHE1 activity in myocyte determined from the pH_i_ recovery following a uniformly-applied ammonium prepulse. Note the magnitude of flux is lower because NHE1 activity in half of the myocyte is blocked with DMA (*N* = 14 treated with NOC12; *N* = 12 control experiments from three animals). High [NO] delivered by NOC12 remotely activated NHE1 (*P* < 0.0001, two-way ANOVA). (*E*) Remote signalling between NO and NHE1 was absent in cells pretreated with the GC inhibitor ODQ (5 µM), indicating a role for the diffusible messenger cGMP.

One possible mechanism through which NO may be regulating NHE1 is S-nitrosylation or S-nitrosation. Indeed, many protein targets have been proposed to undergo this post-translational modification,^16^^,^^18^^,^^37^ although the physiological significance of some of these observations has been questioned.^51^ To test whether sarcolemmal NHE1 is substrate for direct NO reactions, immunoblotting using the so-called TMT-method^52^ was performed on membrane fractions of cardiac tissue treated with either high or low [NO], delivered by Langendorff-perfusion with 5 µM NOC12 or 300 µM NOC12 plus 100 µM CPTIO, respectively (Supplementary material online, *Figure S7A*). There was no evidence of any increase in nitros(yl)ation at the band corresponding to NHE1 in response to low or high [NO] (*Figure 4A*), arguing against S-nitros(yl)ation. As a positive control, an increase in nitros(yl)ation was observed in whole-cell lysates subject to treatment with S-nitrosoglutathione (Supplementary material online, *Figure S7B*). The lack of NHE1 S-nitros(yl)ation by high levels of [NO] was confirmed using the complementary technique of organo-mercury resin-assisted capture that is considered a more specific test for S-nitros(yl)ated residues (SNO)^53^ (*Figure 4B*). Blots were quantified as the ratio of SNO signal to input (NHE1), and this analysis revealed no significant difference between control and NOC12-treated hearts (*Figure 4C*). As confirmation of the method’s resolving power to detect changes in protein nitros(yl)ation, lysates pretreated with DTT or GSNO produced measurable changes on blots (Supplementary material online, *Figure S8*).

The lack of evidence for a direct S-nitros(yl)ation of NHE1 suggests the involvement of cGMP signals and is consistent with the ODQ-sensitivity of NO actions on NHE1 (*Figure *3E* and **G*). If the diffusible messenger cGMP were responsible for the coupling between NO and NHE1, then a spatially-confined source of NO would be expected to influence the activity of more remote NHE1 targets. However, with increasing distance from NO-activated GC, the concentration of cGMP will decay as a result of degradation by phosphodiesterases (PDEs). Since NO exerts a biphasic effect on NHE1, spatially-decaying [cGMP] may flip from being an inhibitory signal to becoming activatory. To test for remote signalling by NO, a dual microperfusion system was used to deliver two sharply separated microstreams at a perpendicular angle to a myocyte. One microstream contained NOC12 to release NO, whilst the other contained CPTIO to scavenge any NO spill-over (*Figure 4D*). The fluorescent dye DAR-4M reported a rise in [NO] that was spatially restricted to the NOC12-exposed half of the cell (*Figure 4E*). Thus, using dual microperfusion, it is possible to deliver NO to one side of a myocyte, and observe any remote actions on NHE1 at the opposite end.

To trigger NHE1 activity, myocytes were first acid-loaded, by means of ammonium prepulse, and then dually microperfused. One half of the myocyte was exposed to NOC12 at a dose (5 µM) that would inhibit NHE1, if applied uniformly to a cell (*Figure 3E*). To investigate how this localized source of NO influences NHE1 remotely, the NOC12-containing microstream also included DMA to inactivate NHE1 locally; consequently, any pH_i_ recovery produced by the cell must be attributable to NHE1 activity at the other half of the myocyte. Measurements of NHE1 flux showed a stimulatory effect of NOC12 when the donor was delivered at a distance from NHE1 (*Figure 4F*). This remote NO-NHE1 coupling was absent in cells pre-treated with ODQ, indicating the involvement of diffusible cGMP signals (*Figure 4G*). Thus, high [NO], which would locally inhibit NHE1, produces an activatory effect on more remote NHE1 protein. This flipping of polarity can be explained by a [cGMP]-dependence of action: low [cGMP] is activatory, whilst high [cGMP] is inhibitory.

### 3.6 Crosstalk between cGMP and cAMP produces biphasic modulation of NHE1 by NO

Based on the results of *in vitro* studies using His182 (*Figure 1*), the pattern of NHE1 phosphorylation produced by PKG is distinct to that evoked by PKA. Thus, it is possible to explain biphasic NO regulation of cardiac NHE1 by dose-dependent recruitment of kinases. PKG was found to be more potent at phosphorylating Ser703 (linked to activation; *Figure 1E*) than Ser648 (linked to inhibition; *Figure 1F*), in comparison to PKA which acted strongly on Ser648.^54^ Notwithstanding this *in vitro* evidence, it is not intuitive to explain how a NO signal, acting through its canonical second messenger cGMP, could orchestrate NHE1 activation at low concentrations, but inhibition with stronger stimulations. A plausible mechanism by which a cGMP signal could switch its polarity of action may involve the recruitment of cAMP beyond a threshold [cGMP]. Indeed, previous studies have shown that cGMP-evoking stimuli can produce a secondary rise in [cAMP], arising from the inhibitory effect of cGMP on the cAMP-degradating enzyme phosphodiesterase-3 (PDE3).^55^

**Figure 5:**
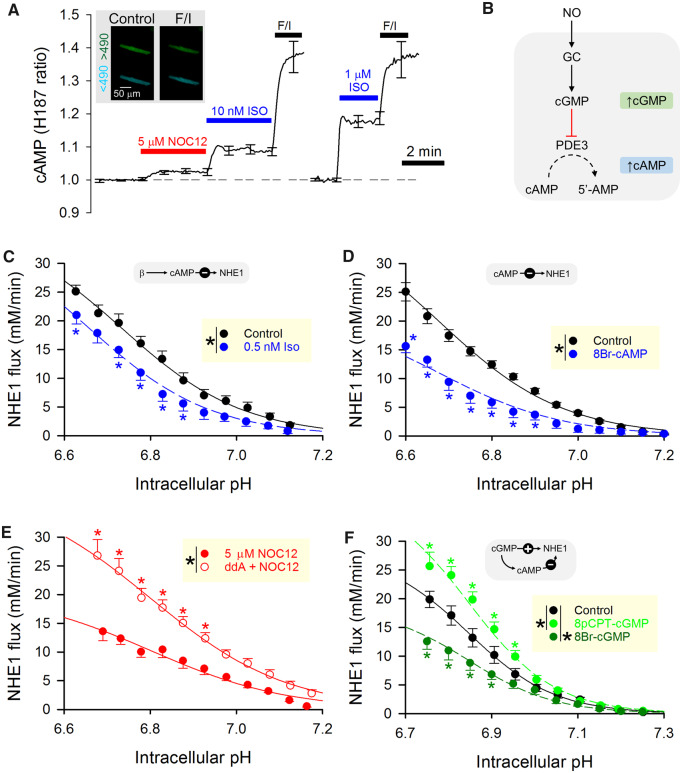
Biphasic regulation of NHE1 by NO is achieved by balancing cGMP-cAMP crosstalk. (*A*) Rat myocyte infected with H187, a FRET-based sensor of cAMP (inset). NOC12 (5 µM) produced a significant increase in signal. As a positive control, F/I (forskolin and IBMX) raises cAMP to levels that saturate the sensor. Isoprenaline (ISO) is a cAMP-mobilizing β-agonist. NO release from NOC12 produces a rise in [cAMP] equivalent to the effect of ∼1 nM Isoprenaline (Iso). (*B*) Proposed mechanism through which cGMP increases [cAMP]. (*C*) Effect of 0.5 nM Iso on NHE1 flux (*N* = 10 Iso-treated cells from three animals; *N* = 19 controls from three animals), showing significant reduction in flux (*P* < 0.0001; two-way ANOVA). (*D*) Effect of 20 µM 8Br-cAMP on NHE1 flux (*N* = 15 treated cells from four animals; *N* = 15 control cells from four animals), showing significant reduction in flux (*P* < 0.0001; two-way ANOVA). (*E*) Effect of 5 µM NOC12 when adenylyl cyclase activity is inactivated with membrane-permeable inhibitor dideoxyadenosine (ddA; *N* = 40 myocytes from five animals). NHE1 flux was significantly greater if cAMP production was ablated (*P* < 0.0001; two-way ANOVA). (*F*) Effect of two different membrane-permeable cGMP analogues, 8pCPT-cGMP and 8Br-cGMP, on NHE1 flux vs. control (*N* = 18, 21, 18 cells from four animals). 8pCPT-cGMP (which does not mobilise cAMP) increased NHE1 activity (*P* < 0.0001; two-way ANOVA), whereas 8Br-cGMP (which evokes a secondary cAMP signal) decreased NHE1 activity (*P* < 0.0001; two-way ANOVA).

To determine if the cGMP signal evoked with 5 µM NOC12 is sufficient to produce a detectable rise in [cAMP], myocytes were first infected with virus containing the gene for H187, a FRET-based cAMP probe. Superfusion with 5 µM NOC12 produced a measurable rise in [cAMP], reported as a response that was equivalent to the effect of low-dose (∼1 nM) isoprenaline (Iso), a beta-agonist (*Figure 5A*). Thus, a sufficiently large NO signal can evoke a cAMP response in adult myocytes (*Figure 5B*).

The effects of cAMP on NHE1 were tested in wild-type rat myocytes stimulated with either 0.5 nM Iso or 20 µM 8Br-cAMP, a membrane-permeable cAMP derivative. Engaging cAMP-dependent signalling with these agents produced an acute inhibition of NHE1 (*Figure *5C and *D*), consistent with previous studies showing a PKA-dependent phosphorylation of the inhibitory site at Ser648.^3^ Thus, a cAMP signal emerging from a NO-evoked rise in [cGMP] may explain the net inhibitory effect of high [NO] on NHE1. To test this, myocytes were exposed to 5 µM NOC12 to activate cGMP signalling, but one group of cells was pretreated with 50 µM dideoxyadenosine (ddA), a membrane-permeable inhibitor of adenylyl cyclase, which is expected to prevent the secondary [cAMP] rise. NHE1 activity was significantly higher in myocytes that were blocked from evoking a cAMP signal, representing the state of NHE1 modulation in response to cGMP alone (*Figure 5E*).

The coupling between NO-NHE1 was further interrogated by introducing membrane-permeable cGMP derivatives in the absence of an NO donor. The analogue 8Br-cGMP activates canonical cGMP targets, such as PKG, and also inhibits PDE3. In contrast, 8pCPTcGMP, unlike endogenous cGMP, is not broken down by PDEs and also does not inhibit PDE3.^56^ When applied to myocytes, 20 µM 8Br-cGMP and 10 µM 8pCPTcGMP produced opposite effects on NHE1 flux (*Figure 5F*). The activatory effect of 8pCPTcGMP, which is unable to evoke secondary cAMP signals, was consistent with the actions of low [NO] (*Figure 3G*), whereas the effect of 8Br-cGMP is more akin to that of high [NO] (*Figure 3E*). Thus, the biphasic effect of NO on NHE1 is a result of a [NO]-dependent rise in cGMP, which *per se* is activatory on NHE1, but switches to becoming inhibitory when a sufficient degree of PDE3 inhibition allows [cAMP] to build-up. This mechanism exploits the distinct patterns of NHE1 phosphorylation produced by PKG and PKA (*Figure 1*).

### 3.7 NO control of NHE1 activity produces a biphasic effect on Ca^2+^ wave frequency

**Figure 6:**
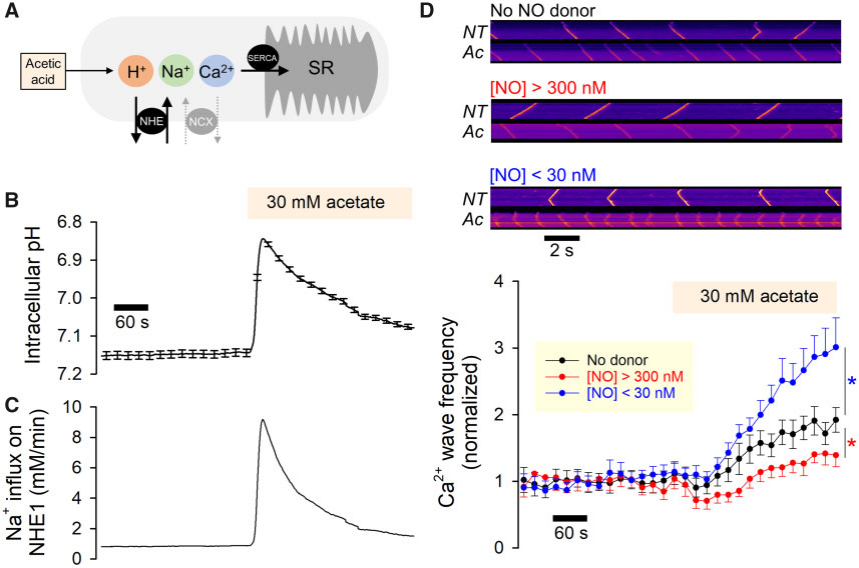
Biphasic regulation of NHE1 by NO affects the propensity of aberrant forms of Ca^2+^ signalling evoked by mild acidosis. (*A*) Schematic model of mechanism linking mild acidosis (attained with 30 mM acetate) to sarcoplasmic reticulum (SR) overload. (*B*) Rat myocytes (loaded with pH-sensitive cSNARF1) under superfusion with Hepes-buffered normal Tyrode with [Ca^2+^] raised to 5 mM to produce Ca^2+^ overload and increase the incidence of waves. After a 6 min period in control solution (NT, normal Tyrode), cells were exposed to 30 mM acetate (Ac) which acidifies the myocyte and activates NHE1 (13 cells from two animals). (*C*) Predicted Na^+^ influx on NHE1, based on pH-sensitivity of flux determined in *Figure 2-2*, mapped onto the pH_i_ time course. (*D*) Rat myocyte loaded with Ca^2+^ dye Fluo3, imaged in line scan mode. Exemplar line scans are shown for a cell in control conditions, and after 6 min of superfusion with 30 mM acetate. Line scan measurements repeated on cells stimulated with either high or low [NO] released from 5 µM NOC12 (producing [NO]>300 nM) or with 300 µM NOC12 plus 100 µM CPTIO (producing [NO]<30 nM), respectively. Results presented as Ca^2+^ frequency normalized to baseline level (*N* = 22 for control, 13 for high [NO], 13 for low [NO]; from five animals). Low [NO] increased Ca^2+^ wave frequency (*P* < 0.0001; two-way ANOVA), whereas high [NO] decreased Ca^2+^ wave frequency (*P* < 0.0001; two-way ANOVA).

The triggering of NHE1 activity at low pH_i_ evokes a substantial influx of Na^+^ ions, in the range of several mM/min, which can Ca^2+^-overload the cardiomyocyte through a well-documented mechanism involving NCX (*Figure 6A*). Beyond a threshold of Ca^2+^ loading, myocytes will produce spontaneous Ca^2+^ release from the sarcoplasmic reticulum (SR), detected as cytoplasmic Ca^2+^ waves. Modulation of Na^+^-influx via NHE1 by NO signals is therefore expected to influence the frequency of spontaneous Ca^2+^ waves.

To trigger NHE1 activity, the cytoplasm of myocytes was uniformly acidified by exposure to 30 mM acetate, which enters and acidifies the cytoplasm and triggers the activation of NHE1 (*Figure 6B*). As NHE1 attempts to extrude the acid-load, Na^+^ ions enter the myocyte (*Figure 6C*). Ca^2+^ release events were recorded in Fluo3-loaded myocytes, superfused with solutions containing 5 mM Ca^2+^ (to raise the likelihood of spontaneous release). The frequency of Ca^2+^ waves was calculated from the interval between events detected by imaging in linescan mode (*Figure 6D*). In the absence of NO donors, Ca^2+^ wave frequency increased by a factor of two within 5 minutes of acidification (*Figure 6D*). The protocol was repeated in the presence of either low [NO] (300 µM NOC12 plus 100 µM CPTIO) to stimulate NHE1 or high [NO] (5 µM NOC12) to inhibit NHE1. It is well-established that NO can affect Ca^2+^ waves through various NHE1-independent mechanisms (e.g. on RyR2 channels),^37^^,^^57^^,^^58^ and to correct for these actions, the response of Ca^2+^ wave frequency to acetate was normalized to baseline frequency. In the presence of high [NO], the acetate-evoked rise in Ca^2+^ wave frequency was attenuated (to only ∼150% of resting levels), consistent with an inhibitory effect on NHE1-dependent Na^+^-influx (*Figure 6D*). Thus, inhibition of NHE1 with high doses of NO is anti-arrhythmogenic, similar to the pharmacological effect of NHE1 inhibitors such as cariporide.^59^ In contrast, low [NO] resulted in a more robust acetate-evoked rise in Ca^2+^ wave frequency (to ∼300% of resting levels), explained by the larger Na^+^ influx through activated NHE1 (*Figure 6D*).

## 4. Discussion

We provide the first report of a physiologically relevant molecule exercising a *biphasic* effect on cardiac NHE1, the heart’s most powerful pH_i_ regulator and major Na^+^ entry pathway. NHE1 activity has a broad remit of actions on cardiac physiology because its activity influences pH_i_ homeostasis and Ca^2+^ signalling, and thence a myriad of downstream effects. Although resting pH_i_ in myocytes is normally kept near 7.2, deviations can occur pathologically, such as during ischaemia, or physiologically in response to intra- or extracellular signals.^54^^,^^60–62^ Controlled shifts in pH_i_ are implemented physiologically by changing transmembrane H^+^ ion traffic carried by transporters, such as NHE1. Unlike other regulators of NHE1, NO does not operate through a surface receptor, and thus the coupling to its downstream enzyme (GC) has less ‘gain’ than in the case of G protein-coupled cascades. Instead, the physiological outcomes of NO signalling are fine-tuned by regulating the pattern of gas release. Other examples of dose-dependent actions of NO on the heart have been reported for chronotropy,^63^^,^^64^ but these are the ensemble of effects on various proteins, and not necessarily a convergence onto a single identifiable target.

The regulatory scope of NO signalling on NHE1 can be quantified from the dynamic range of its effect, calculated as the ratio of the highest to the lowest activation state. Based on results such as those in *Figure 3*, the dynamic range for the NO effect on NHE1 is a factor of two, offering good leverage on cardiac physiology. At the lower end of this activity range, NHE1 is still able to maintain a low intracellular [H^+^] that is conducive for cellular physiology, but this reduction in homeostatic prowess would permit some degree of pH_i_ fluctuations, serving as *bona fide* H^+^-signals within the so-called permissive range.^1^ When maximally activated, cardiac NHE1 can produce an unprecedented magnitude of H^+^ extrusion to defend against acid-challenges and maintain an alkaline pH_i_.

Intriguingly, the biphasic effect of NO on NHE1 was not observed in neonatal rat ventricular myocytes or in a breast cancer cell line (Supplementary material online, *Figure S6*). This finding indicates that the biochemical details of the NO-NHE1 cascade cannot be resolved in expression systems (e.g. with the use of NHE1 mutants) or with cell-free approaches. Here, we were able to obtain mechanistic insights by studying the responses of primary adult myocytes to pharmacological or genetic manipulations of the putative NO-NHE1 cascade (*Figures 2*–*6*), and interpreting these findings in terms of how PKA and PKG differentially phosphorylate the C-terminus of NHE1, as determined using *in vitro* assays (*Figure 1*).

**Figure 7:**
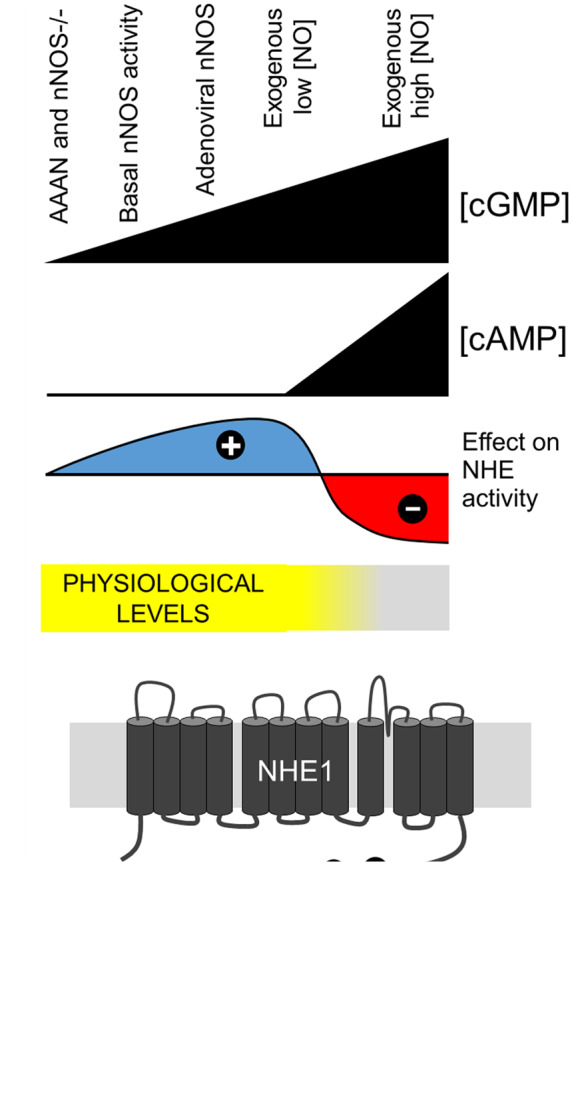
Model of cardiac NHE1 regulation by NO signalling. (*A*) The various experimental manoeuvres performed in this study are ranked by strength of NO signal. Higher NO stimulations will evoke greater cGMP production. At higher [cGMP], inhibition of PDE3 permits a steady rise in [cAMP]. The balance between cGMP and cAMP signals sets the level of NHE1 activity. Based on literature values for [NO] measured *in vivo*, NO-evoked NHE1 activation is expected to be within physiological signalling, whereas NO-evoked NHE1 inhibition would occur over the pathophysiological range of elevated [NO]. (*B*) Schematic of the signalling pathways, evoked by NO, that regulate NHE1 activity by means of a differential activation of PKG-and PKA-dependent cascades.

The modulation of NHE1 activity by NO is not exercised by S-nitros(yl)ation, tested biochemically by two independent methods (*Figure **4**A*–*C*), but through the enzyme GC instead (*Figures 3 and **4*) Canonically, this pathway signals through the diffusible messenger cGMP, but in the heart, adequate cGMP-inhibition of PDE3 can allow a secondary rise in [cAMP], which we confirmed with a FRET-based sensor of cAMP (*Figure 5A*). Results of *in vitro* studies (*Figure*1*A*–*C*) show that serine residues of the regulatory C-terminus of NHE1 are substrates for cAMP- and cGMP-dependent kinases. Using antibodies against specific phosphorylation sites, we show that PKA and PKG have distinct selectivity for serine residues. Specifically, PKG showed a preference for phosphorylating Ser703, a residue linked to NHE1 activation. PKA, in contrast, was able to produce a strong phosphorylation of Ser648. Our functional data explain how NO signals are able to use these kinases to orchestrate NHE1 activation and inhibition at low and high [NO], respectively, by recruiting cGMP in a dose-dependent manner, and triggering cAMP only with the higher concentration (*Figure 7*). Using pharmacological inhibition and knockdown, we show that basal NO production by nNOS was activatory on NHE1. Adenoviral delivery of nNOS, which preferentially targets the sarcolemma,^65^ or a carefully titrated low (<30 nM) dose of NO (NOC12+CPTIO) produced a further stimulatory effect (*Figure 7A*).^44^ In the latter experiments, NO concentrations attained inside myocytes are likely to be attenuated further by chemical reactions involving proteins such as myoglobin (∼200µM myoglobin will scavenge NO with a rate constant of 22 µM/s^66^). We therefore argue that the low [NO] concentration delivered by mixing NO donor and scavenger is within the physiological range.^26^ NHE1 activation by this level of NO signalling could be explained by PKG phosphorylation of Ser703, without affecting Ser648 (*Figure 1E*). Exposure to higher [NO] (>300 nM) released from donors (SNP or NOC12 alone) switched the polarity of NO signalling to net inhibition because of the secondary recruitment of cAMP signalling (*Figure 3*). Activation of PKA is expected to increase Ser648 phosphorylation, an inhibitory modification (*Figure 1F*). Indeed, a previous report showing NHE1 inhibition by NO released from a high dose of SNP^21^ is likely to have involved cAMP signals (*Figure 5B*). Thus, the crossover in the polarity of the NO-NHE1 cascade occurred around 100 nM, postulated to be above the normal physiological range of NO signalling.^26^ As a test of our model, a cGMP-analogue that cannot inhibit PDE3 (and hence has no effect on [cAMP]) was found to activate NHE1 (*Figure 5F*), whereas stimuli that evoked cAMP signals but without a change in cGMP, caused NHE1 inhibition (*Figure **5**C* and *D*). Furthermore, it was possible to reverse the effect of a nominally inhibitory NO signal (i.e. domain of high [NO]) by imposing a diffusion distance between the source of NO and NHE1, along which the cGMP signal decays and becomes inadequate to raise [cAMP] (*Figure 4*).

Given that the actions of NO on NHE1 are highly context-dependent, as shown by their manifestation in adult but not neonatal myocytes (*Figure 3*, Supplementary material online, *Figure S6*), it is plausible that pathologic remodelling of myocytes may alter NO-NHE1 coupling further. Patterns of myocardial NO signalling can undergo changes in heart failure,^67–69^ hypertension,^70^ and ischaemia/reperfusion injury^68^^,^^71^^,^^72^ as a result of altered NOS isoform expression^67^^,^^68^^,^^70^^,^^71^ and subcellular distribution^67^^,^^68^ or abnormal intracellular chemistry (e.g. NO scavengers^73^ and NOS co-factors^19^) In failing hearts, for instance, raised nNOS activity at the sarcolemma^67^^,^^68^ may produce a more intensive release of NO near NHE1, potentially resulting in inhibition.

The tightening of pH_i_ control by NO-activated NHE1 would improve the responsiveness of the myocyte’s pH_i_-regulatory apparatus to acidic disturbances, such as in periods of increased metabolic demand or failure of perfusion. However, the benefit of this augmentation must be weighed against the additional energetic burden on the Na^+^/K^+^ ATP pump required to restore the inward transmembrane [Na^+^] gradient. The NHE1-driven increase in intracellular [Na^+^] will also affect Ca^2+^ signalling by increasing SR Ca^2+^ loading. At best, this would exert a positive inotropic effect, but more sustained overloading will eventually precipitate an arrhythmia as a result of spontaneous Ca^2+^ release. Considering the injurious nature of the latter phenomenon, there is a biological niche for agents that protect against NHE1 over-drive. High volumes of NO release would produce such an effect, as shown by the inhibitory effect on Ca^2+^ wave frequency (*Figure 6D*). We speculate that basal-to-low levels of NO serve to increase heart contractility by stabilizing pH_i_ at a more alkaline level, and by increasing cellular Ca^2+^ loading through increased Na^+^ entry. However, excessive activation of NHE1 can be acutely deleterious to the heart, as demonstrated by ischaemia–reperfusion injury^74^ when cells become Ca^2+^ overloaded^11^ and arrhythmic.^75^ Chronically overactive NHE1 has been linked to maladaptive hypertrophy, a risk factor in heart failure.^76^^,^^77^ Thus, enhanced NO signalling may serve as a natural NHE1 inhibitor, producing more quiescent Ca^2+^ signalling by curtailing Na^+^ influx and allowing the cell to undergo a mild acidification.

Modulation of NHE1 by NO signalling may be relevant to the interpretation of clinical trials, such as the GUARDIAN trial (cariporide)^78^ because the benefit of targeting NHE1 will depend on the extent to which the protein is functionally active. Patients and animal models with HFpEF (heart failure with preserved ejection fraction) have greatly increased nitrosative stress^69^ which may be equivalent to levels of NO that inhibit NHE1. In these states, pharmacological inhibition of NHE1 would yield a reduced therapeutic effect, and drugs such as cariporide may not be as effective as anticipated. Compared to HFpEF, myocardial ischaemia is postulated^72^ to involve more modest increases in NO levels. If such a nitrosative stress activates NHE1, a higher dose of cariporide would be required to adequately suppress Na^+^ influx. This may explain why the sole beneficial outcomes noted in the GUARDIAN trial were for the highest doses (120 mg) of cariporide.^78^ However, without information about the state of NO signalling, it is not possible to perform a meta-analysis of patients stratified by endogenous NHE1 activity.

In summary, the biphasic effect of NO provides unprecedented flexibility in controlling NHE1 locally in the heart, and with it, the capacity to modulate cardiac Ca^2+^ signalling and other pH-sensitive processes.

## Supplementary material

Supplementary material is available at *Cardiovascular Research* online.

## Supplementary Material

cvz311_supplementary_data

## Notes

Translational perspective Na^+^/H^+^ exchanger-1 (NHE1) regulates intracellular [H^+^] and [Na^+^], but its over-activation can drive ionic imbalances that affect cardiac contractility and rhythm. Pharmacological control of NHE1 (e.g. with cariporide) has been proposed as cardioprotective in conditions such as ischaemia/reperfusion injury, but trials (e.g. GUARDIAN) failed to demonstrate overall clinical benefit. A confounding factor in these analyses is NHE1 modulation by endogenous factors. We demonstrate that nitric oxide (NO) signalling fine-tunes NHE1, producing stimulation at low levels, turning into inhibition as the signal grows stronger. Thus, evaluations of the therapeutic efficacy of NHE1-blocking drugs should consider NO signalling, a pathway known to undergo changes in disease.
