# Supplementary material for: Nitric oxide modulates cardiomyocyte pH control through a biphasic effect on sodium/hydrogen exchanger-1
Source: Cardiovasc Res. 2019 Nov 19;116(12):1958–71. doi: 10.1093/cvr/cvz311 (PMC7567331; doi:10.1093/cvr/cvz311)
Supplement: cvz311_supplementary_data [file cvz311_supplementary_data.docx]

SUPPLEMENT

**SUPPLEMENTARY METHODS**

***In vitro kinase reactions.*** Phosphorylation of His182 was conducted using commercial *in vitro* kinase systems for PKA (V4246; Promega, USA) and PKG (V4248; Promega, USA). Samples for Phos-tag analysis and conventional western blotting were generated using reactions containing 500ng His182, 200ng kinase, 50µM ATP, 50 µM DTT, and for PKG reactions, 10 µM cGMP, in a final volume of 25 µl. Reactions were incubated at room temperature for the relevant time point until termination by the addition of SDS-PAGE loading buffer: Laemmli reducing buffer for western blotting or LDS reducing buffer for Phos-tag analysis. Kinase reactions for mass spectrometry contained 10 µg His182, 1mM ATP, 50 µM DTT and 5 µg PKA or 2.5 µg PKG with 10 µM cGMP, in a final volume of 45 µl, incubated at room temperature for 120 minutes. These reactions were terminated by addition of 20mM EDTA before being snap frozen in liquid nitrogen for subsequent analysis.

***Membrane protein extraction.*** Sprague-Dawley rats (300-325g) were euthanized as stated earlier and whole hearts were perfused on a Langendorff apparatus solution for 15 mins, during which treatments were applied. Hearts were snap-frozen in liquid N_2_ and stored at -80°C until required. Membrane fractions were obtained using the Thermo Scientific Mem-PER Plus Membrane Protein Extraction Kit (Thermo, 89842). Briefly, 100 mg cardiac samples from adult hearts were homogenised in a permeabilisation buffer containing inhibitors of proteases and phosphatases and 100µM neocuproine (to inhibit enzymatic cleavage of S-nitrosocysteines) to lyse cells and release cytosolic proteins into solution. Samples were then centrifuged to pellet the membrane fraction, the supernatant, containing cytosolic proteins, were collected. Pellets were resuspended in a solubilisation buffer to solubilise membranes, before being centrifuged again. The supernatants, containing membrane proteins, were collected. Cytosolic and membrane fractions were aliquoted and stored at -80°C until required. Protein samples were quantified using a BCA protein assay kit (Thermo, 23227).

***Western blotting.*** Membrane protein samples or His182 peptide were prepared using 4x Laemmli sample buffer (with 2-mercaptoethanol). Membrane proteins were then resolved by SDS poly-acrylamide gel electrophoresis (SDS PAGE) prior to transfer to a PVDF membrane. The membranes were blocked in 5% non-fat milk in Tris-buffered saline containing 0.2% Tween-20 (TBS-T) for 1 hour at RT before incubation with primary antibody overnight at 4°C. Secondary antibodies diluted in TBS-T were incubated with membranes for 1 hour at RT before imaging using ECL (Li-Cor WesternSure® Premium Chemiluminescent Substrate) with a Bio-Rad ChemiDoc™ Imaging System.

***Phos-tag.*** For Phos-tag gel analysis of His182 phosphorylation, a Bis-Tris– buffered neutral pH gel system was adopted. For this system, samples were added to 2x lithium dodecyl sulfate loading buffer (282 mM Tris base, 212 mM Tris HCl, pH 8.5, 20% glycerol, 4% lithium dodecyl sulfate, 200 µM DTT, 1 mM EDTA, 0.66 mM SERVA Blue G250, and 0.35 mM Phenol Red) to give a 1x final concentration. Samples were heated to 95°C for 5 min and centrifuged at 10,000 rpm for 2 min. Samples were separated by hand-cast 12% Bis-Tris PAGE mini-gels that were supplemented with 50 µM Phos-tag acrylamide and 100 µM Zn(NO_3_)_2_ with a 4% stacking gel. Gels were electrophoresed at 140 V via the stacking gel for 5 min and 120 V via the resolving gel. Gels were washed in transfer buffer containing 1mM EDTA for 10 minutes with gentle agitation, then washed in transfer buffer without EDTA for 10 minutes with gentle agitation prior to transfer to PVDF membrane.

***S-nitros(yl)ation (iodoTMT) assay.*** For detection of protein S-nitrosocysteine post-translational modifications, S-nitros(yl)ated cysteines of membrane proteins were labelled with a modified biotin switch assay that used iodoacetyl tandem mass tag (iodoTMT; Qu et al, J Proteome Res 2014) for labelling instead of biotin-HPDP (Jaffrey & Snyder SH, Sci STKE 2001) to reduce background during Western blot detection (Thermo, 90105). MMTS was added to 100 μg protein samples to block free cysteine thiols. Proteins were precipitated and excess MMTS removed using acetone at -20°C. Precipitated samples were resuspended in RIPA buffer containing inhibitors of proteases and phosphatases and 100µM neocuproine (to inhibit enzymatic cleavage of S-nitrosocysteines). Proteins were then labelled with iodoTMT prior to Western blotting. For positive and negative controls, protein samples were incubated with S-nitrosoglutathione or reduced glutathione, respectively.

***Organo-mercury enrichment of S-nitros(yl)ated proteins.*** Phenylmercury resin was used to capture NO-Cys containing proteins (Doulias et al, Methods 2013). Following enrichment, bound proteins were eluted and the extent of NHE S-nitros(yl)ation assessed by immunoblotting. For positive and negative controls, lysates were incubated with 10 µM S-nitrosoglutathione or 10 mM DTT, respectively.

***Antibodies:***

| *Blot* | *Primary Antibody* | *Secondary Antibody* |
| --- | --- | --- |
| TMT | Mouse anti-TMT, 1:1000; Thermo 90075 | Goat anti-mouse HRP, 1:10,000; Thermo 626520 |
| NHE1 | Mouse anti-NHE1, 1:500; BD Biosciences 611775 | Goat anti-mouse, 1:5,000; Thermo 62-6520 |
| GAPDH | Anti-GAPDH HRP-conjugate, 1:10,000;Proteintech HRP-60004 | N/A |
| Na^+^/K^+^ ATPase | Rabbit anti-Na^+^/K^+^ ATPase α, 1:500; Santa Cruz Biotechnology sc-28800 | Goat anti-rabbit, 1:10,000; Thermo 656120 |
| pSer | Rabbit anti-phospho-Serine, 1:500; Cell Signaling Technologies #2981 | Goat anti-rabbit, 1:5,000; Thermo 656120 |
| pAkt | Rabbit anti- phospho-Akt substrate; 1:500; Cell Signaling Technologies #9614 | Goat anti-rabbit, 1:5,000; Thermo 656120 |
| 14-3-3 | Rabbit anti-phospho-(Ser) 14-3-3 Binding Motif; 1:500; Cell Signaling Technologies #9601 | Goat anti-rabbit, 1:5,000; Thermo 656120 |

***Mass spectrometry MALDI.*** For MALDI (Matrix Assisted Laser Desorption/Ionization Time of Flight) mass spectrometry analysis one µL of each sample (100 ng/µl His182 in PKA 50 ng/µl and His182 100 ng/µl with PKG, 50 ng/µl) was mixed with 1 µL of sinapinic acid (10 mg/ml in 50% acetonitrile/water + 0.1% trifluoroacetic acid). One µL of the sample/matrix solution was then spotted onto a stainless-steel target plate and allowed to air dry. Mass spectra were obtained using a Bruker Autoflex Speed MALDI-ToF (Bruker Daltonic GmbH). Ions were analyzed in positive mode and external calibration was performed by use of a standard protein mixture.

***Analysis of Tryptic Peptides of Control and Phosphorylated His182.*** Trypsin digestion was performed on control and phosphorylated His182 protein. Briefly, samples were dried, dissolved in 100 mM ammonium bicarbonate to make a His182 concentration of 0.2 µg/µl, and 1 µg of each solution was then digested with trypsin (40 ng trypsin (Promega sequencing grade) in 5 ul) at 37^o^C overnight. The samples were then dried, dissolved in 40 µL of water + 0.2% formic acid and 5 µl was injected for analysis. Tryptic peptides were resolved and ionized by using nanoflow HPLC (Easy-nLC II, Thermo Scientific) with a PicoFrit fused silica capillary column (New Objective ProteoPepII, C18, 100 μm ID, 300 Å, 5 μm) coupled to an LTQ XL-Orbitrap hybrid mass spectrometer (Thermo Scientific). Samples were injected onto the column at a flow rate of 3000 nL/min and resolved at 500 nL/min using a 60 minute linear gradient from 0 to 35% v/v aqueous acetonitrile in 0.2% v/v formic acid. The mass spectrometer was operated in data dependent acquisition mode using external mass calibration, with a resolution of 30,000 and *m*/*z* range of 400–2000. The fourteen most intense multiply charged ions were sequentially fragmented by using collision induced dissociation, and spectra of their fragments were recorded in the linear ion trap. Data was processed using Proteome Discoverer 1.4 (Thermo Scientific) and searched against the supplied sample sequence using SEQUEST (Thermo Scientific). Search parameters included a precursor mass tolerance of 10ppm and a fragment mass tolerance of 0.8 Da with oxidized methionine and deamidated asparagine and glutamine as dynamic modifications.

**SUPPLEMENTARY FIGURES**

| 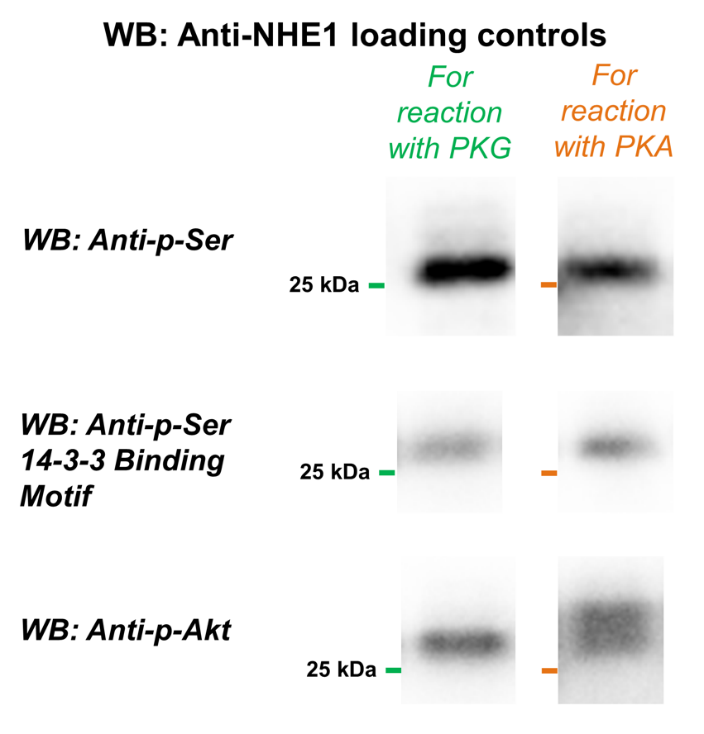 | **Supplementary Figure S1:** Loading controls for blots presented in Fig 1D/E/F. |
| --- | --- |

| 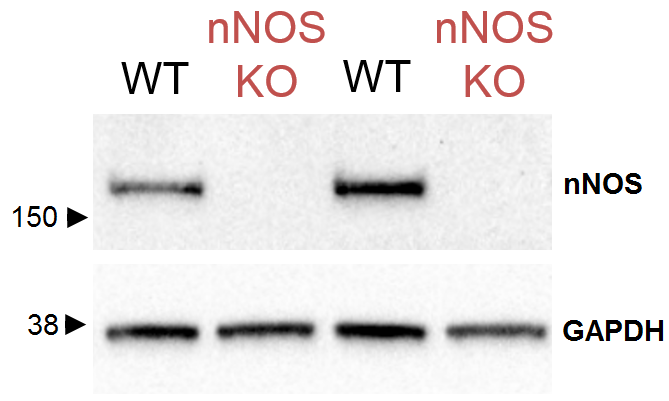 | **Supplementary Figure S2:** Confirmation of nNOS knockout in left ventricle lysates. Protein extracted from LV tissue with 20ug loaded per lane. Anti-mouse nNOS (A-11 clone, Santa Cruz); 1:500 in 5% BSA/PBST. HRP-conjugated GAPDH (Promega); 1:25,000 in 1% BSA/PBST |
| --- | --- |

| 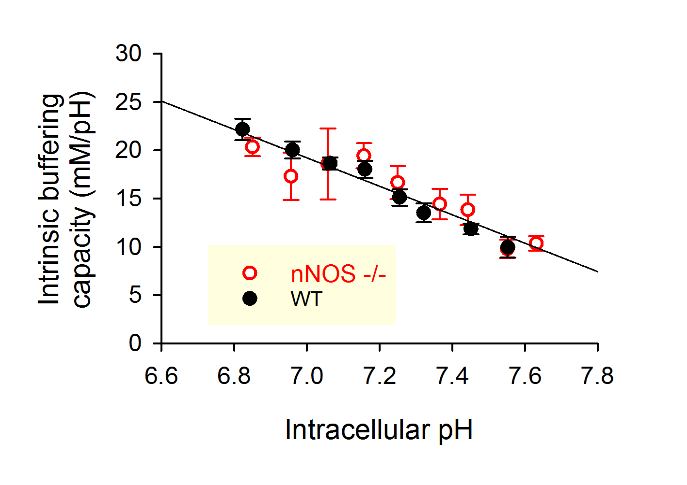 | **Supplementary Figure S3:** Intrinsic buffering capacity determined in Hepes-buffered superfusates in myocytes isolated from nNOS-/- mice (20-35 cells from 5 animals) or their wild-type littermates (30-40 cells from 5 animals). Data obtained using the stepwise ammonium removal protocol. Best fit: line of regression. |
| --- | --- |


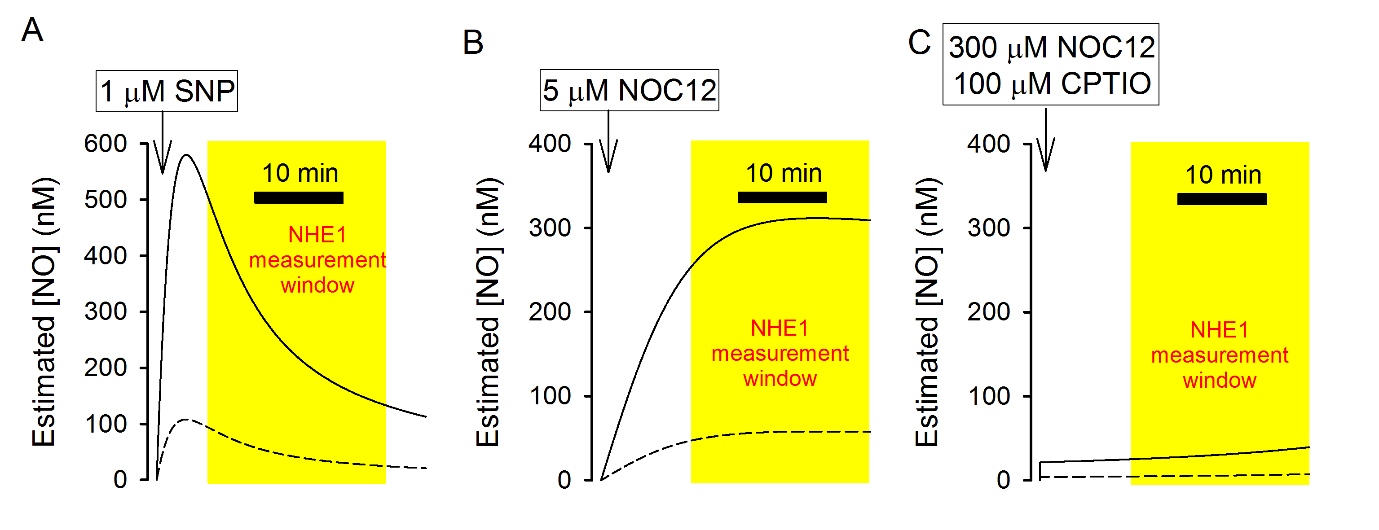


**Supplementary Figure S4:** *Mathematical prediction of the time-course of NO release from donors.* Mathematical model (Griffiths, Wykes, Bellamy & Garthwaite, *Mol Pharmacol* 2003; 64: 1349-1356) was used to predict the time course of [NO] produced by NO donors. Measurements of NHE1 activity were typically made during the period shaded yellow. Continuous line shows predicted [NO] in the superfusate delivered to myocyte. Dashed line shows predicted cell-averaged intracellular [NO], assuming a cylindrical cell of radius 12 µm and a length constant of [NO] decay of 0.6 µm, calculated assuming that myoglobin (Mb) is the primary NO scavenger inside cells, and that neither NOC12 nor CPTIO permeate the cell membrane. Length constant calculation: square root of (D_NO_/k_Mb_×[Mb]), where D_NO_ is NO diffusivity (3.3×10^-5^ cm^2^/s), k_Mb_ is the binding constant of NO to Mb (22×10^6^ µM^-1^ s^-1^) and [Mb] is the Mb concentration (200 µM). **(A)** NO release from 1 µM SNP (half-life 1.5 min at 37 deg C). **(B)** NO release from 5 µM NOC12 (half-life 100 min at 37 deg C). **(C)** NO release from 300 µM NOC12 in mixture with NO-scavenger CPTIO (100 µM) and urate (300 µM).

| **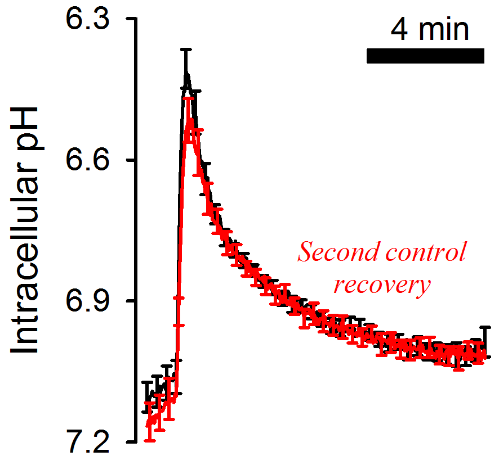** | **Supplementary Figure S5:** Two consecutive pH recovery time courses obtained from paired experiments (ammonium prepulse). NHE1 activity is stable and does not show a time-dependent degradation. Average of 10 cells from 2 animals. |
| --- | --- |


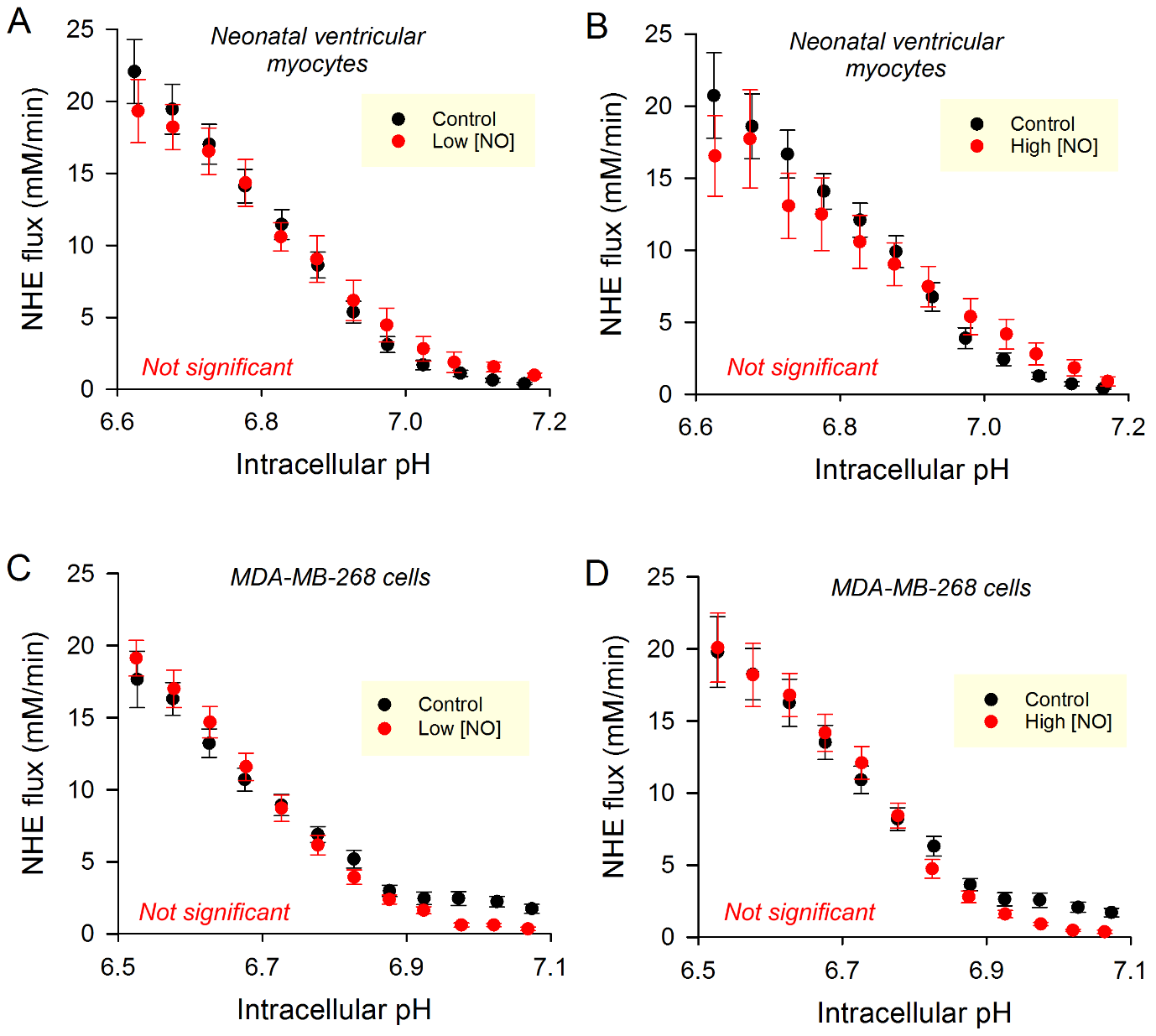


**Supplementary Figure S6:** *The biphasic effect of NO is a special feature of the adult myocyte signalling environment.* Experimental protocols performed on cultured neonatal rat ventricular myocytes. Effect of **(A)** low (300 µM NOC12 + 100 µM CPTIO) and **(B)** high (5 µM NOC12) [NO] on NHE activity (N=25-40 cells from 4 isolations, each of 10-12 pups). No significant effects were observed. Experimental protocols were repeated on the breast cancer cell line MDA-MB-468 which produces a similar magnitude of NHE1 flux to adult myocytes. Effect of **(C)** low (300 µM NOC12 + 100 µM CPTIO) and **(D)** high (5 µM NOC12) [NO] on NHE activity (55-60 cells). No significant effects were observed.


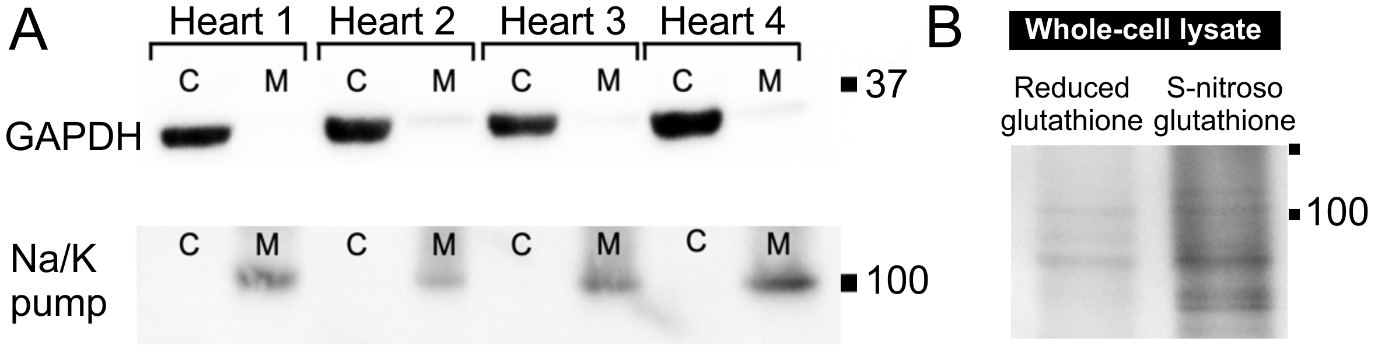


**Supplementary Figure S7:** *Immunoblotting.* **(A)** Left ventricular tissue was lysed and processed to separate membrane fraction, M, from cytoplasmic fraction, C. The membrane fraction was confirmed by Na^+^/K^+^ pump alpha subunit (but not GAPDH) immunoreactivity; the cytoplasmic fraction was confirmed by GAPDH (but not Na^+^/K^+^ pump) immunoreactivity. **(B)** Whole-cell lysate obtained from ventricle tested by antibody raised against TMT. The protocol for S-nitros(yl)ation was performed, in which lysates were treated with either reduced glutathione or its S-nitroso derivative; the latter increases protein S-nitros(yl)ation. An overall increase in S-nitros(yl)ation was observed.

| **Supplementary Figure S8:** *Organo-mercury enrichment.* To confirm adequate detection of changes in protein SNO using this method, lysates from biological duplicates were treated with either DTT (to fully reduce SNO modifications) or GSNO (to induce SNO) and then underwent organo-mercury enrichment followed by SDS-PAGE. Coomasie staining showed a complete reduction of protein SNO in DTT treated samples, while GSNO increased the extent of total protein SNO relative to control hearts. | 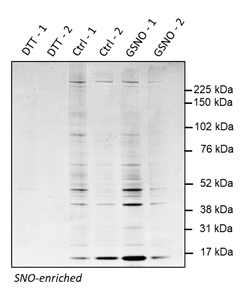 |
| --- | --- |
